# Coronary artery abnormalities in Kawasaki disease according to patient age

**DOI:** 10.1186/1546-0096-9-S1-P86

**Published:** 2011-09-14

**Authors:** Esmeralda Nuñez, Patricia Martínez, Victorio Cuenca, Francisco Jesus García, David Moreno, Antonio Urda

**Affiliations:** 1 Carlos Haya Children Hospital, MÁLAGA, Spain

## Background and aims

Kawasaki disease (KD) is considered the leading cause of acquired heart disease in childhood in developed countries. Despite diagnose this disease is difficult in the case of infants, it is required a high clinical suspicion to ensure an early treatment. The aim of this study is compare clinical presentation and rate of coronary artery abnormalities (CAA) according to children age.

## Methods

Retrospective analysis of patients diagnosed with KD from January 2006 to April 2011. Epidemiological, clinical and laboratory characteristics, also when the treatment was initiated and the how was the outcome for younger and older than 12 months.

## Results

35 patients were diagnosed with KD, 13 younger than 1 year (37.1%). 54.3% of them met the classical criteria, while 45.7% were considered incomplete presentation: fever +3 criteria (31.4%) and fever +2 criteria (14.3%), it was more common in children under 12 months (53.8%). Clinical manifestations more frecuent associated with fever were: rash (97.2%), oropharyngeal involvement (94.3%) and conjunctival injection (74%). Laboratory abnormalities are shown in the table. Every case was treated with immunoglobulin, 29 in the first 7 days of fever (82.8%) and 6 later on (17%); fever resolved within 24 hours in 65.7% patients and 48 hours in 20%. Only 4 children required a second dose (3 younger than 12 months). 84.6% of patients younger than 12 months developed CAA, 2 of them with aneurysms. Rate of CAA in over 12 months patients was 18.2% and nobody developed aneurysms. According to the onset time of treatment, 41.4% of patients treated in the first week developed CAA and 50% of patients treated later. Rate of gallbladder hydrops was higher in under 12 months patients (15.4%). Serious outcomes were developed in a 4 months patient with coronary aneurysms up to 6 mm, cardiorespiratory arrest caused by ventricular dysfunction and distal ischemia of limbs which required the removal of several phalanges.

## Conclusions

Higher rate of coronary involvement in KD infants younger than 12 months, which is more common in those treated after 7 days from onset of fever. Acute-phase reactants such as CRP or PCT could be viewed as coronary artery abnormalities predictors on larger studies. Therefore, cardiac ultrasound should be considered in infants with unexplained prolonged fever and elevated acute phase reactants, even though not meeting all criteria.

